# Chitosan as Valuable Excipient for Oral and Topical Carvedilol Delivery Systems

**DOI:** 10.3390/ph14080712

**Published:** 2021-07-23

**Authors:** Szymon Sip, Magdalena Paczkowska-Walendowska, Natalia Rosiak, Andrzej Miklaszewski, Katarzyna Grabańska-Martyńska, Karolina Samarzewska, Judyta Cielecka-Piontek

**Affiliations:** 1 Department of Pharmacognosy, Poznan University of Medical Sciences, 4 Swiecickiego Street, 60-781 Poznan, Poland; szymonsip@ump.edu.pl (S.S.); mpaczkowska@ump.edu.pl (M.P.-W.); nrosiak@ump.edu.pl (N.R.); 2 Institute of Materials Science and Engineering, Poznan University of Technology, Jana Pawła II 24, 61-138 Poznan, Poland; andrzej.miklaszewski@put.poznan.pl; 3 Department of Internal Medicine, Poznan University of Medical Sciences, Grunwaldzka 16/18, 60-786 Poznan, Poland; kgrabanska@wp.pl; 4 Department of Clinical Auxiology and Pediatric Nursing, Poznan University of Medical Sciences, Szpitalna 27/33 Street, 60-572 Poznan, Poland; ksamarzewska@ump.edu.pl

**Keywords:** chitosan, carvedilol, oral and topical delivery system, bioadhesion, permeability

## Abstract

Chitosan is a valued excipient due to its biocompatibility properties and increasing solubility of poorly water-soluble drugs. The research presented in this paper concerns the preparation of binary combinations of chitosan (deacetylated chitin) with carvedilol (beta-blocker) to develop a formulation with a modified carvedilol release profile. As part of the research, six physical mixtures of chitosan with carvedilol were obtained and identified by spectral (PXRD, FT-IR, and Raman), thermal (DSC), and microscopic (SEM) methods. The next stage of the research estimated the profile changes and the dissolution rate for carvedilol in the obtained drug delivery systems; the reference sample was pure carvedilol. The studies were conducted at pH = 1.2 and 6.8, simulating the gastrointestinal tract conditions. Quantitative changes of carvedilol were determined using the developed isocratic UHPLC-DAD method. Established apparent permeability coefficients proved the changes in carvedilol’s permeability after introducing a drug delivery system through membranes simulating the gastrointestinal tract and skin walls. A bioadhesive potential of carvedilol–chitosan systems was confirmed using the in vitro model. The conducted research and the obtained results indicate a significant potential of using chitosan as an excipient in modern oral or epidermal drug delivery systems of carvedilol.

## 1. Introduction

Chitosan is a polysaccharide obtained mainly as an effect of the N-deacetylation of chitin [1,2]. It is one of the most abundant renewable natural chemicals in the world of nature, occurring naturally, being the building blocks of living organisms such as crustaceans, mollusks, and insects. Chitosan exhibits cationic properties due to protonated amino groups that can bind negatively charged substances such as polymers or anionic forms of active pharmacological substances present in the form of salts. Due to such interaction, chitosan is one such excipient, which can modify the pharmaceutical properties of active pharmaceutical ingredients (API), such as apparent solubility and dissolution rate [3,4,5]. In recent years, chitosan has become the center of the pharmaceutical field interest due to its favorable biological properties such as biocompatibility, lack of toxicity, and biodegradability when administered orally due to the influence of the intestinal microflora [2,6]. Chitosan has confirmed antibacterial, antiviral, and antifungal properties. Chitosan can induce wound healing, improve the body’s immune response in contact with pathogens, and strengthen the anti-inflammatory effect [7,8].

Due to its properties, chitosan can be used as a solubilizer, improving the dissolution of drugs. The solubility of about 70% of the latest pharmaceutical oral compounds in the early stage of research is less than 100 µg/mL and is often a limitation in achieving the required bioavailability. Therefore, it is a critical stage in the development of new drug delivery systems. The rate of drug dissolution in the gastrointestinal tract can significantly determine the bioavailability of poorly water-soluble drugs, especially drugs belonging to the 2nd class of the Biopharmaceutical Classification System (BCS), including carvedilol (CVD). Drugs belonging to this group are characterized by bioavailability, limited only by solubility, due to their high permeability through biological membranes.

CVD is an aryloxyalkylaminopropranol derivative (beta-blockers), which antagonizes the β1- and β2-adrenoreceptors and limits the sympathetic nervous system’s activity. It is one of the most commonly prescribed drugs for cardiovascular diseases such as hypertension, coronary heart disease, or cardiac insufficiency. CVD is a non-cardioselective beta-blocker to dilate blood vessels. At higher doses, it can block calcium channels [9]. The beta-blockers mechanism is the competitive displacement of catecholamines (noradrenaline and adrenaline) from binding sites in β-adrenergic receptors [10,11,12].

Due to the low solubility and high permeability through cell membranes of CVD, its solubility is a critical parameter that allows improving bioavailability. Many technological approaches are used to enhance the dissolution rate, including particle size reduction to increase the surface area, solubilization in surfactant system, formation of water-soluble complexes, usage of a prodrug, and manipulation of solid-state of drug substance to improve drug dissolution, i.e., by decreasing drug crystallinity [3,13]. One of the widely developed topics is drug delivery systems preparation using natural carriers, e.g., chitosan [14,15]. An appropriate matrix allows for the successful improvement of CVD’s therapeutic efficacy and minimizes conventional treatment therapy’s side effects. The CVD release profile from nanoparticles showed a biphasic release pattern with an initial burst release in the first 2 h followed by a controlled release throughout 72 h, with higher bioavailability than marketed tablet formulation in phosphate buffer saline (PBS) pH 7.4 [14]. Chitosan nanoparticles using a spray-drying method were characterized by prolonged and controlled release, which may provide an increased clinical value compared to conventional formulations, which results from the reduced dosing frequency and prolonged therapeutic effect as well as increased bioavailability [16]. Except for oral tablets, intranasal carvedilol–chitosan microspheres with good mucoadhesive properties were able to increase drug absorption and consequently significantly improve the carvedilol bioavailability [17].

The second idea to overcome CVD’s low absorption from the gastrointestinal tract is transdermal delivery [18]. Besides, drugs belonging to beta-blockers (timolol and propranolol) are registered to treat hemangiomas in newborns. Topical administration is relatively well tolerated and has few side effects while maintaining a high therapeutic response [19,20]. The use of beta-blockers in topical administration allows for use in the treatment of glaucoma [21].

Transdermal administration is one of the recent trends in developing drugs chronically with low bioavailability of high first-pass effect to maintain optimal therapeutic concentration. Applying APIs onto the skin allows for local action and delivers a drug into the systemic circulation across the skin. This drug delivery route mechanism has many advantages, including steady drug plasma concentrations, improved patient compliance, elimination of hepatic first pass, and degradation in the gastrointestinal tract [22]. The literature indicates various attempts to approach transdermal administration of CVD, from patches to gels and polymer nanoparticles loaded with the active substance [23,24,25,26,27,28,29].

In chronic diseases, patient compliance is essential, often determined by the possibility of a single administration of the active substance. The use of chitosan as an excipient offers such potential. The prolonged release of the active substance from the group of beta-blockers or their administration across the skin also eliminates vasospasm resulting from the entire therapeutic dose’s administration in a short time. The prolonged release of beta-blockers from oral delivery systems or transdermal administration reduces the undesirable effects associated with saturation in antagonizing adrenergic receptors (hyperglycemic effect).

This study aimed to prepare CVD systems with an excipient (chitosan). First, chitosan’s influence on changes in CVD dissolution rate and permeability through model systems of artificial membranes (PAMPA) simulating the walls of the gastrointestinal system and skin was assessed. In parallel, the adhesive strength of the prepared chitosan system was investigated.

## 2. Results

The conducted tests are the basis for determining chitosan use as an excipient for sustained release in the oral carvedilol (CVD) delivery system and the transdermal system. Interactions in the CVD system developed during the tests had a significant effect on the solubility and permeability. Changes in CVD concentration during solubility and permeability studies were controlled using developed and validated HPLC-DAD methods (Table S1, Supplementary Materials). The conducted research allowed us to determine the bioadhesive potential of the obtained systems.

The CVD was mixed with two chitosans of different viscosity 80/500 and 80/1000 in three different weight ratios (1:1, 1:5, and 1:10 *w*/*w*). The grinding method was chosen to obtain chitosan systems because it allows obtaining micronized particles of sparingly soluble API to increase the solvent’s contact surface [30]. The method is cheap, easy to perform, and there are no severe conditions (temperature, pH, or pressure) that may affect the API. In the literature, kneading is described as one of the key and most frequently used methods of obtaining complexes [15,31,32].

**Figure 1:**
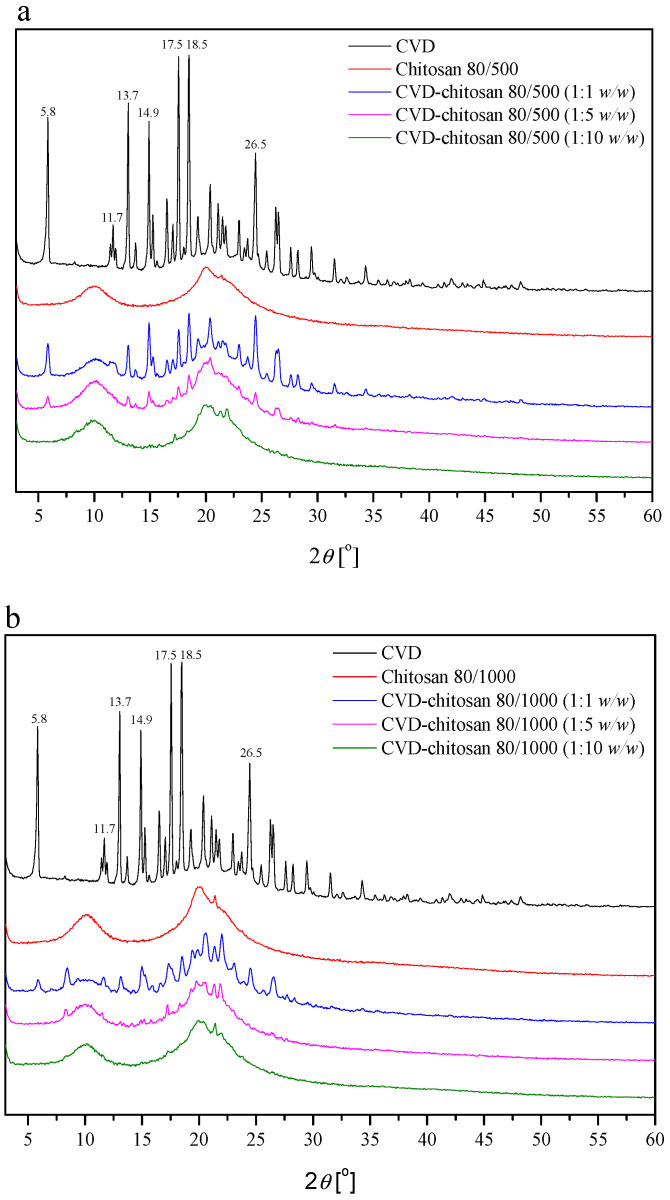
Powder X-ray diffractograms of the solid samples with chitosan 80/500 (**a**) and chitosan 80/1000 (**b**) 2*θ* positions of the principal diffraction peaks are shown for CVD.

Using spectral methods, such as PXRD, FT-IR, and Raman spectroscopy identification, made it possible to define intramolecular interactions between compounds in received systems. The first method of assessing interactions in the resulting systems was PXRD. It was revealed that CVD was crystalline and that the starting material powder was the trihydrate form, as evidenced by the position of diffraction peaks at 5.8°, 11.7°, 13.7°, 14.9°, 17.5°, 18.5°, and 26.5° 2*θ* (Figure 1) [17]. PXRD patterns of chitosan starting material exhibited two crystalline reflections at around 10° and 20° 2*θ*, which confirmed the literature data [33]. The degree of crystallinity values of CVD decreased with the increase in polymer concentration [3].

**Figure 2:**
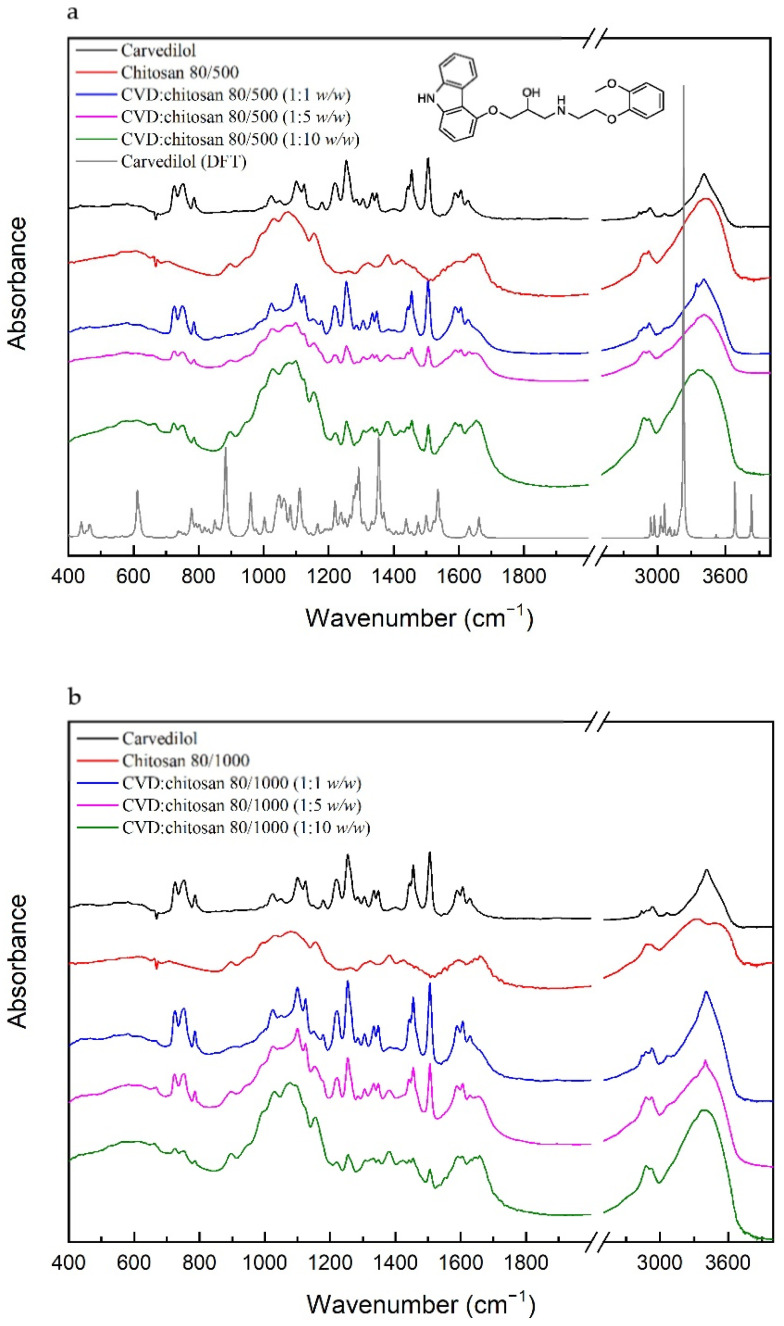
The IR absorption spectra of the solid systems of CVD-chitosan 80/500 (**A**) and CVD-chitosan 80/1000 at room temperature (**B**).

Calculation and experimental IR absorption spectra of CVD based on the computational methods, which characterize individual bonds in absorption spectra and FT-IR spectra of the solid systems of CVD with chitosan, are displayed in Figure 2.

For the CVD, most characteristics are the bonds associated with the C–C, C=C, C–O, C–N, N–H, or O–H bonds. The bonds related to the stretching vibration of the C=C bonds in the ring in 3-[2-(2-methoxyphenoxy)ethylamino]propan-2-ol and 1-(9H-carbazol-4-yloxy) part of the molecule are located at 1589 and 1605, 1629 cm^−1^, respectively, while the bonds corresponding to the stretching vibration of the C–C bonds are observed at 1100, 1124, 1304, 1334, 1455, and 1504 cm^−1^. The first two of them have additional components related to the stretching vibration of the C–O bonds in 1-(9H-carbazol-4-yloxy) and the rocking vibration of the C–H bonds in all molecules. The bond at 1304 cm^−1^ corresponds to the twisting vibration of the C–H bonds in 3-[2-(2-methoxyphenoxy)ethylamino]propan-2-ol bond at 1455 cm^−1^, which are related to the scissoring and rocking vibration of the C–H bonds. The last bond has an additional mod related to the stretching vibration of the C–N and C–O bonds in 1-(9H-carbazol-4-yloxy) and the rocking vibration of the C–H bonds in the 1-(9H-carbazol-4-yloxy) part of the molecule. The stretching vibration of the C–C bonds is also related to the bonds described as breathing rings, for example, the bond at 1347 cm^−1^. This bond is also associated with the bending vibration of the C–N–C bonds and the rocking vibration of the C–H bonds in 1-(9H-carbazol-4-yloxy). The next type of vibration represents the bonds at 1178, 1254, and 1400 cm^−1^, and they are mainly related to the stretching vibration of the C–N bonds. They have an additional component corresponding to the twisting and wagging vibration of the C–H bonds in all molecules, bending vibration of the C–O–H bonds in 3-[2-(2-methoxyphenoxy)ethylamino]propan-2-ol, and bending vibration of the P–O–H bonds in the phosphate group. Bonds related to the bending vibration of the C–O–H bonds are located at 1220 and 1285 cm^−1^, while the P–O–H’s bending vibration is located at 1023, 1049, and 1220 cm^−1^. The two bonds of them at 1023 and 1049 cm^−1^ have additional components associated with the stretching vibration of the C–C and C–O bonds in 3-[2-(2-methoxyphenoxy)ethylamino]propan-2-ol and 1-(9H-carbazol-4-yloxy) and rocking vibration of the C–H bonds. In contrast, the bond related to the stretching vibration of the P–O is located at 752 cm^−1^. The bond corresponding to the O–H bonds’ deformation is located at lower frequencies (788 cm^−1^), and the bonds related to the stretching vibration of these bonds are observed at above 3400 cm^−1^. Between 2800 and 3400 cm^−1^ are located the bonds corresponding to the stretching vibration of the C–H bonds in 3-[2-(2-methoxyphenoxy)ethylamino]propan-2-ol (2837, 2932, and 3065 cm^−1^).

For the chitosan, the 80/500 and the 80/1000 most characteristics, IR absorbance peaks range from 897–1650 cm^−1^ and 2800–3450 cm^−1^ (Figure 2). The FT-IR spectra of chitosan include significant vibrations at 897 cm^−1^ (C–H bending out of plane), 1032 cm^−1^ (C–O stretching), 1074 cm^−1^ (C–O stretching), 1156 cm^−1^ (asymmetric C–O–C stretching), 1258 cm^−1^ (bending vibrations of hydroxyls), 1318 cm^−1^ (C–N stretching of amide III), 1382 cm^−1^ (CH_3_ symmetrical deformation), 1427 cm^−1^ (CH_2_ bending), 1593 cm^−1^ (N–H bending of the primary amide), and 1649 cm^−1^ (C=O stretching of amide I). In the range of 2800–3450 cm^−1^, bands correspond to C–H symmetric and asymmetric stretching vibrations (2875 cm^−1^ and 2919 cm^−1^) and O–H stretching vibrations (3437 cm^−1^). The spectra of chitosan 80/1000 in the range of 3000–3500 cm^−1^ have two components at 3318 cm^−1^ (N–H stretching vibrations) and 3512 cm^−1^ (O–H stretching vibrations). They correspond well with intramolecular hydrogen bonds [17].

The conducted FT-IR test allowed us to determine the lack of new bond formation in the obtained CVD systems with chitosan. In each of the systems, the sum of CVD and chitosan bands are visible. In the spectrum recorded for the CVD-chitosan system (1: 1 *w*/*w*), despite the equal ratio, the bands derived from CVD prevail. For the ratios of 1: 5 and 1:10 *w*/*w*, the spectra assume the character of pure chitosan; however, one can also distinguish bands characteristic of CVD, that is: 727 cm^−1^ (C–H bending outside of the plane in 3-[2-(2-methoxyphenoxy)ethylamino]propan-2-ol and 1-(9H-carbazol-4-yloxy)); 752 cm^−1^, 788 cm^−1^ (P–O stretching and O–H wagging in phosphate, respectively); 1220 cm^−1^ (P–O–H bending in phosphate + C–O–H bending in 3-[2-(2-methoxyphenoxy)ethylamino]propan-2-ol); 1254 cm^−1^ (C–N stretching in 1-(9H-carbazol-4-yloxy) + C–H twisting in 3-[2-(2-methoxyphenoxy)ethylamino]propan-2-ol + P–O–H bending in phosphate); 1443 cm^−1^ (C–H rocking, C–C stretching and C–N stretching in 1-(9H-carbazol-4-yloxy)); 1455 cm^−1^ (C–C stretching in 1-(9H-carbazol-4-yloxy) + C–H scissoring in 3-[2-(2-methoxyphenoxy)ethylamino]propan-2-ol + C–H rocking in all molecule); 1504 cm^−1^ (C–C stretching in 3-[2-(2-methoxyphenoxy)ethylamino]propan-2-ol and 1-(9H-carbazol-4-yloxy) + C–N stretching, C–O stretching and C–H rocking in 1-(9H-carbazol-4-yloxy)); 1589 cm^−1^ (C=C stretching in 3-[2-(2-methoxyphenoxy)ethylamino]propan-2-ol); 1605 cm^−1^ and 1629 cm^−1^ (C=C stretching in 1-(9H-carbazol-4-yloxy)). Compared to the FT-IR spectrum of pure CVD, these bands have lower intensity, and we do not observe their shifts.

**Figure 3:**
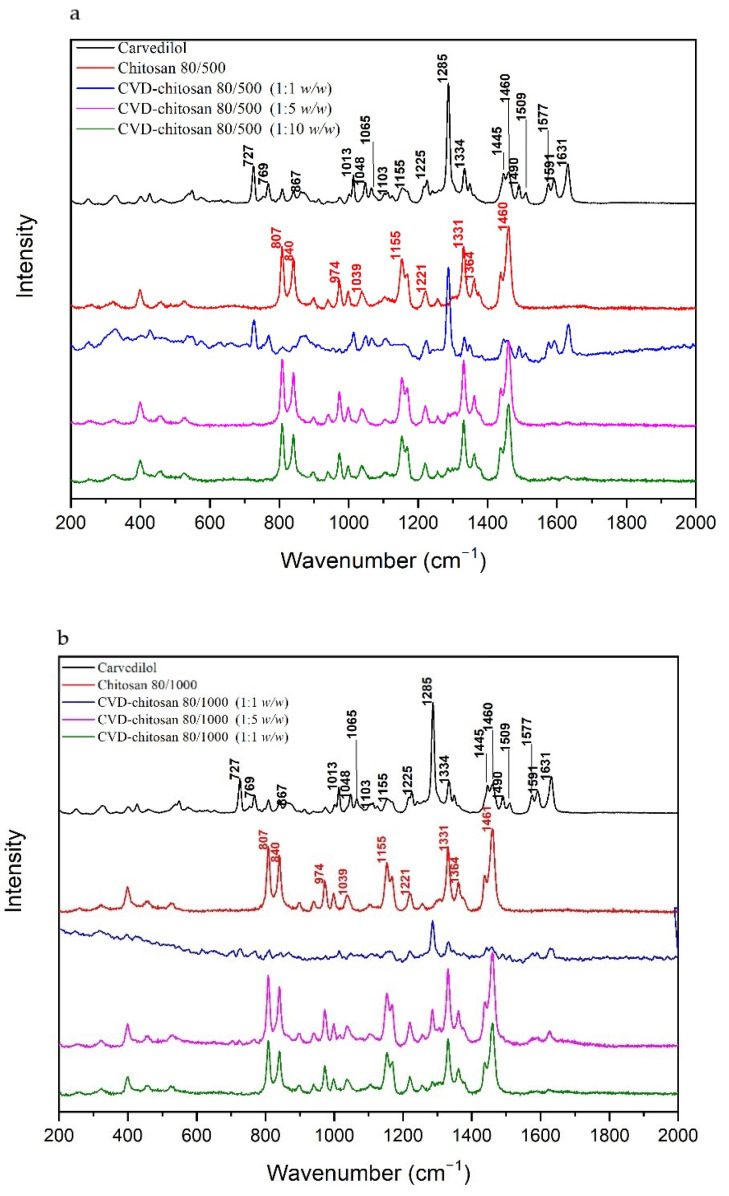
Raman spectra of the solid systems of: CVD-chitosan 80/500 (**A**) and CVD-chitosan 80/1000 (**B**) (λ = 785 nm) at room temperature.

Figure 3 compares spectra obtained at wavelength λ = 785 nm for the pure CVD and c chitosan systems. Most characteristics bands in the CVD Raman spectrum are corresponding to the bending vibration of the C–C–C (727 cm^−1^), N–H (867 cm^−1^, 1225 cm^−1^), C–H (1048, 1103, 1155, 1225, 1509 cm^−1^), and C–N–C (1334 cm^−1^) bonds in 1-(9H-carbazol-4-yloxy). The next type of vibration in 1-(9H-carbazol-4-yloxy) represents the bonds at 1013, 1285, 1460, 1490 cm^−1^ (C–C), 1065 cm^−1^ (C–O), and 1631 cm^−1^ (C=C). In 3-[2-(2-methoxyphenoxy)ethylamino]propan-2-ol, there are bands related to the stretching vibration of the C–O (1013 cm^−1^), C–C (1065 cm^−1^), and C=C (1591 cm^−1^). The bond at 1241 cm^−1^ corresponds to the bending vibrations of the C–O–H bonds and C–H rocking vibrations in 3-[2-(2-methoxyphenoxy)ethylamino]propan-2-ol.

In the Raman spectra for CVD-chitosan 80/500 and CVD-chitosan 80/1000 (1:1 *w*/*w*), bands are derived from CVD. In the case of systems in the weight ratios 1:5 *w*/*w* and 1:10 *w*/*w* predominant bands derived from chitosan. We can distinguish such bands as 807 cm^−1^ (out-of-plane CH deformations of rings), 840 cm^−1^ (C–O–C stretching vibrations), 974 cm^−1^, 1221 cm^−1^, 1331 cm^−1^ (C–H stretching vibrations), 1155 cm^−1^ (C–N stretching vibrations) and 1461 cm^−1^ (CH_3_ bending vibrations) [34].

Just for the CVD-chitosan 80/1000 (1:5 *w*/*w*) system, in which three bands can be distinguished from CVD, i.e., 1013 cm^−1^ (P–O–H bending in phosphate + C–C stretching in 1-(9H-carbazol-4-yloxy) + C–O stretching and breathing ring in 3-[2-(2-methoxyphenoxy)ethylamino]propan-2-ol + C–H rocking in all molecule), 1285 cm^−1^ (C–C stretching in 1-(9H-carbazol-4-yloxy) + C–H twisting in 3-[2-(2-methoxyphenoxy)ethylamino]propan-2-ol), and 1631 cm^−1^ (C=C stretching in 1-(9H-carbazol-4-yloxy)).

**Figure 4:**
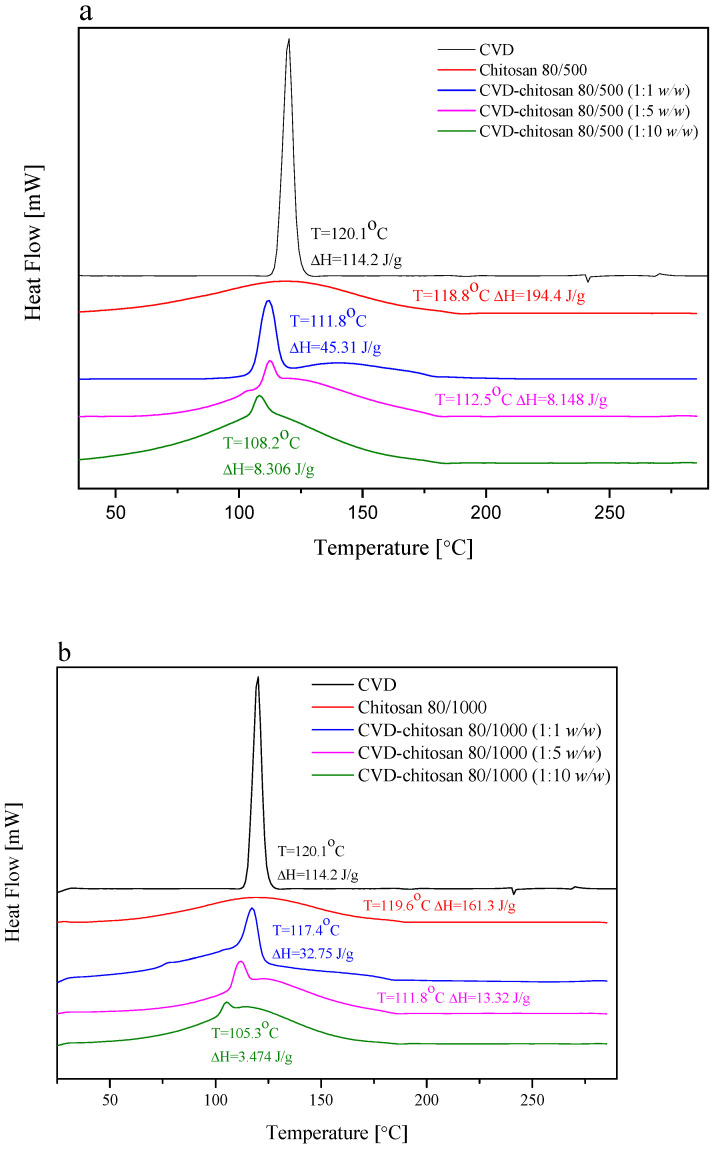
DSC thermograms of the solid systems of: CVD-chitosan 80/500 (**a**) and CVD-chitosan 80/1000 (**b**).

The DSC thermograms collected for chitosan as well as its chitosans systems are presented in Figure 4. The DSC thermograms obtained for pure CVD showed a sharp endothermic peak at 120.1 °C, which is related to the CVD melting point, typical of the anhydrous crystalline drug, whereas the DSC traces of chitosan showed a broad endothermal peak located around 119.0 °C, associated with water evaporation [35]. As a marine polysaccharide in the solid state, chitosan has a strong affinity for water, so the endothermic peak associated with water loss should not be surprising [35]. The second main thermal event registered for chitosans as well as their systems was a decomposition around 300 °C, shown on TGA curves (Figures S2 and S3, Supplementary Materials) [36]. Analyzing thermograms for chitosan-based systems, there was no appreciable change in the melting endotherms in chitosans systems, and the characteristic peak of CVD was well recognized in them at 111.8 °C, 112.5 °C, and 108.2 °C for CVD-80/500 1:1 *w*/*w*, 1:5 *w*/*w*, and 1:10 *w*/*w*, and at 117.4 °C, 111.8 °C, and 105.3 °C for CVD-80/1000 1:1 *w*/*w*, 1:5 *w*/*w*, and 1:10 *w*/*w*, respectively, nearly the same temperature as that of pure drug. The lack of disappearance of this endothermic CVD peak indicated no interaction between CVD and chitosan, which confirms the conclusions obtained on the basis of the spectral analysis [37]. Additionally, those confirmed XPRD patterns where CVD remained in a crystalline form and had not been converted into a fully amorphous state, which was obtained earlier to prepare microspheres [17]. However, when analyzing the enthalpy of individual peaks, it can be seen to be lower than might be expected based on the fraction of the CVD enthalpy (ΔH = 114.2 J/g). This may indicate a reduction in the degree of CVD crystallinity [38], confirmed also based on XPRD patterns, or interaction between CVD and chitosan other than absorption [39], but in the last one, the changes may be so small that they are not visible in the spectra in Figure 2 and Figure 3.

**Figure 5:**
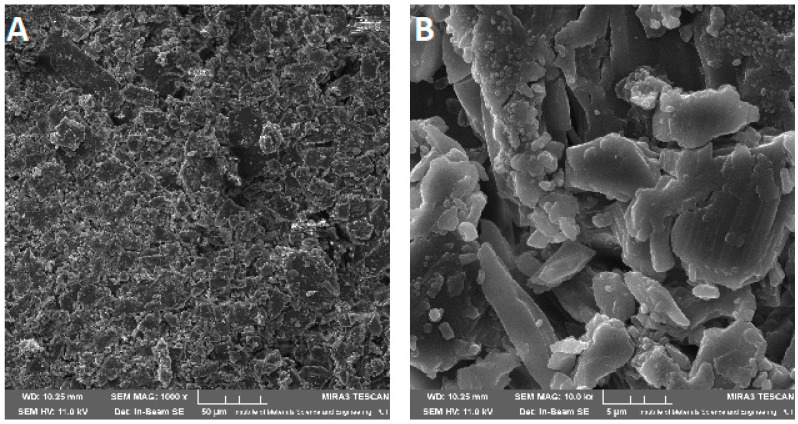
SEM images of CVD at 1000× (**A**) and 10,000× (**B**) magnification.

**Figure 6:**
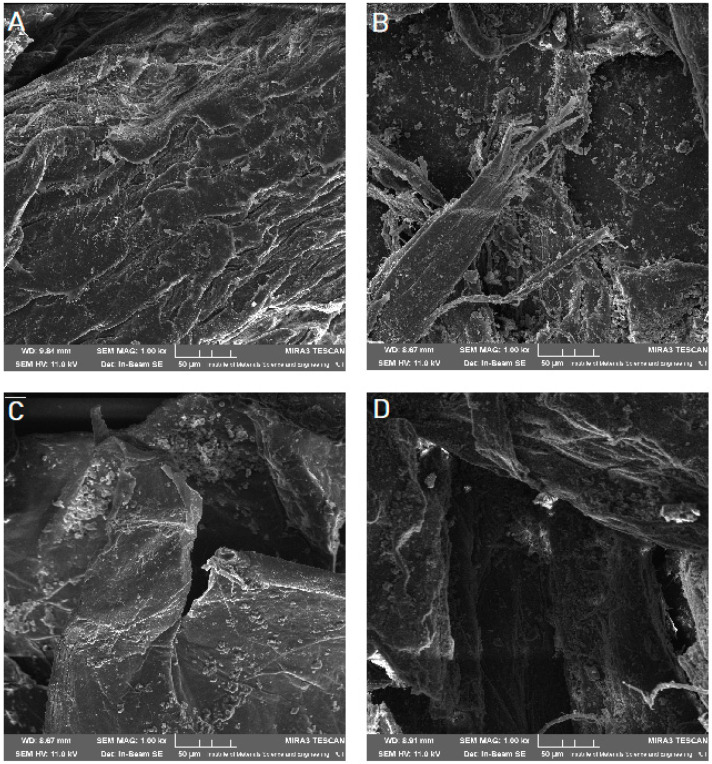
SEM images of chitosan 80/500 (**A**) and its system with CVD in a weight ratio: 1:1 (**B**), 1:5 (**C**), 1:10 (**D**) at 1000× magnification.

**Figure 7:**
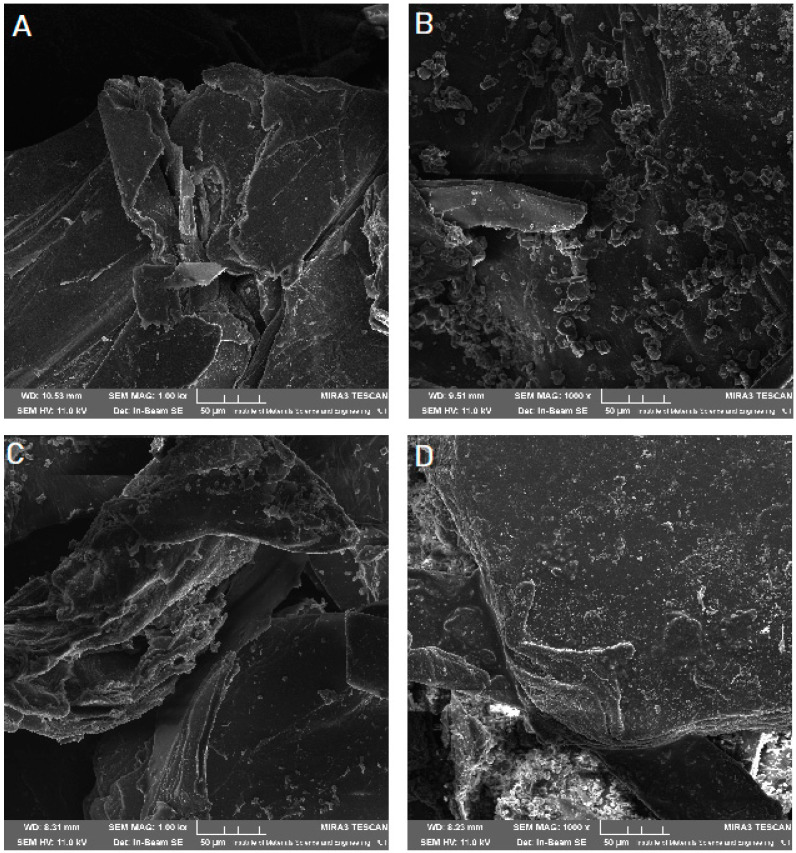
SEM images of chitosan 80/1000 (**A**) and its system with CVD in a weight ratio: 1:1 (**B**), 1:5 (**C**), 1:10 (**D**) at 1000× magnification.

Scanning electron microscope (SEM) imaging was used to determine the surface morphology of the obtained systems. In the photos taken, we observe the crystalline structure of the CVD (Figure 5) with a large grain size discrepancy. The surface morphology of pure chitosans (Figure 6A and Figure 7A) shows a nonporous, smooth, and highly irregular surface with many grooves and depressions constituting the anchor point for API disperse [40]. We do not observe the formation of new structures as a result of the system preparation carried out, only the greater dispersion of CVD on chitosan with the increase in the amount of polymer in the system (Figure 6 and Figure 7B–D), at the same time we observe a reduction in the size of the CVD grains. The reduction of CVD grain size and its dispersion can be attributed to the preparation used; in the kneading process, CVD is micronized on the rigid surface of the chitosan base. The CVD grains obtained in this way are spatially wedged in the irregular, undulating chitosan surface.

**Figure 8:**
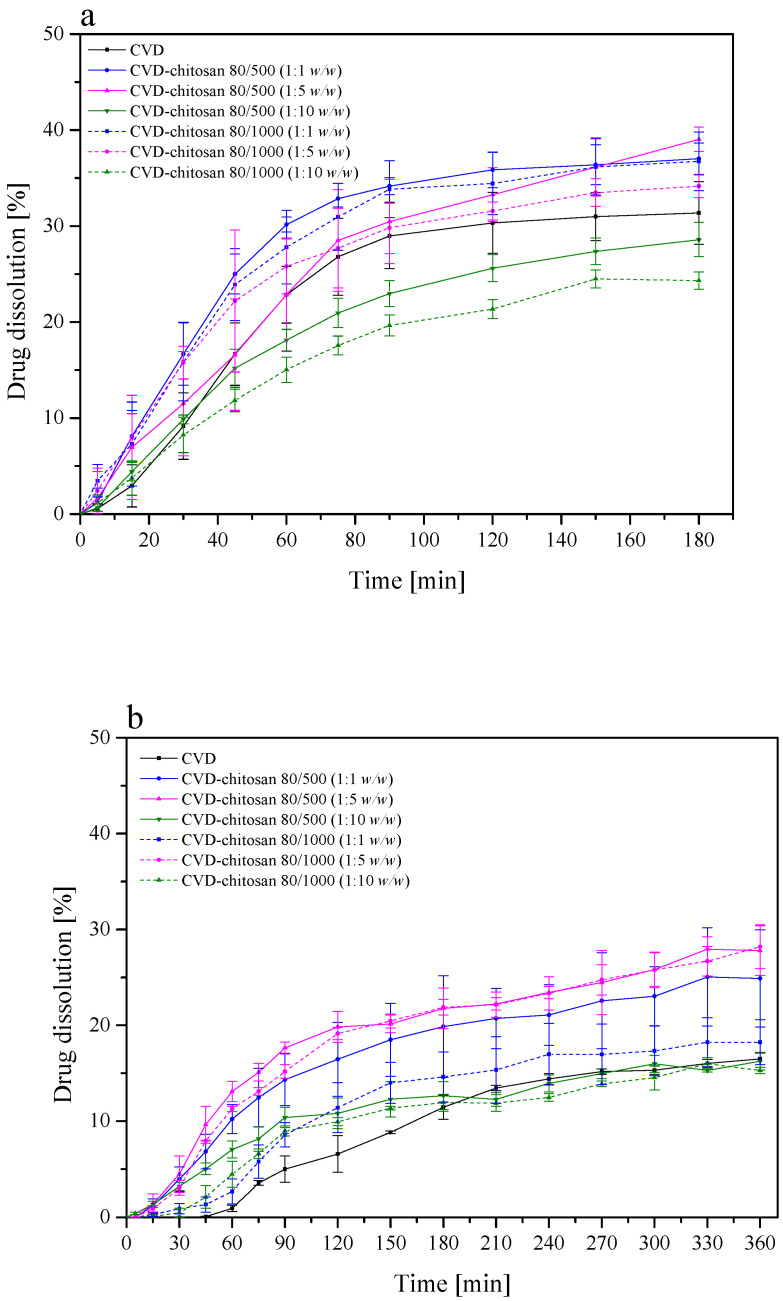
Powder dissolution of CVD from the chitosan systems at pH 1.2 (**a**) and pH 6.8 (**b**).

The dissolution of low solubility and high permeability, Biopharmaceutical Classification Systems (BCS) class II drugs is rate-limiting to oral absorption. Weakly basic BCS class II drugs such as CVD exhibit pH-dependent solubility. It dissolves in the acidic pH of the stomach, as it is presented in its ionized form, while it precipitates in the small intestine. The calculated *f*_1_ and *f*_2_ values confirmed that the CVD-chitosan systems’ dissolution profiles are different from pure CVD (Supplementary Materials, Tables S2 and S3). Moreover, no statistical similarity was demonstrated between the profiles both at pH 1.2 and 6.8. Chitosan and its derivatives are widely described as a good vehicle for enhancing the solubility and dissolution of poorly water-soluble drugs [3,41,42,43]. Chitosan dissolves readily in most of the acid solutions. Upon dissolution, amine groups of the polymer become protonated, resulting in a positively charged polysaccharide (RNH^3+^) and chitosan salts (chloride, glutamate, etc.). The resulting products show solubility in the water. [44]. It can explain better dissolution enhancing properties in acidic conditions. Moreover, literature data have shown that the dissolution rate depends not only on the powder surface and particle size but also on the wettability, which is essential for a hydrophobic drug surface such as CVD. In the previous studies, chitosan also showed increased solubility and dissolution rate of naproxen due to adsorption on the chitosan surface [3]. The spectral analysis showed no interaction between CVD and chitosan, i.e., the adhesion of CVD particles on the chitosan surface confirmed in SEM imaging. Therefore, we can conclude that its adsorption on the chitosan surface increases the solubility of CVD. Due to the dispersion of the API molecules on the uneven surface of the chitosan particles (Figure 6 and Figure 7), we observe an increased wetting surface. In addition to the increased solubility of CVD from the CVD-chitosan system, the sustained release was also noted (Figure 8). Based on dissolution rate profiles (Figure 8) showing the release of CVD from the CVD-chitosan system, it was confirmed that as the percentage of chitosan in the system increases, the dissolution rate of the CVD decreases. Also, the dissolution rate decreased with the higher viscosity of chitosan. During the dissolution process, we observe a significant increase in the volume of chitosan resulting from its gradual wetting by the medium used to create a gel layer on the surface. The resulting layer hinders the escape of CVD particles deposited on the polymer surface, thus limiting the release rate [45]. The resulting layer decreases water entry into the core of the system and, accordingly, may promote a decrease in bioactive dissolution rate [45,46].

CVD, as a BCS II class drug, exhibits low solubility and high permeability [47]. Calculated apparent permeability coefficients of CVD and CVD-chitosan systems at pH 1.2 and pH 6.8 are presented in Table 1. The conducted permeation study was performed for 3 h. The obtained results confirm that the addition of chitosan does not adversely affect the CVD penetration, which both by itself and in combinations, is classified as a high-permeable compound (*P_app_* >1 × 10^–6^ cm/s) [48]. The permeability of CVD from the CVD-chitosan 80/500 1:5 and 1:10 as well as CVD-chitosan 80/1000 1:1 systems using the GIT-PAMPA model was statically higher compared to pure CVD. Similar changes for the CVD-chitosan 80/500 and 80/1000 1:1 systems using the Skin-PAMPA model were observed but were not statistically significant. Despite the high permeation, CVD’s absolute bioavailability is approximately 23% [49].

To overcome the difficulties occurring in oral therapy, there is a need to develop a new drug delivery system to improve therapeutic efficacy. Because of its low dose and extensive hepatic metabolism, CVD is a suitable candidate for topical administration [50,51]. To examine the applicability of the prepared CVD-chitosan systems, permeability through the skin barrier using the PAMPA Skin test was executed. CVD, as well as its chitosan systems, are well-permeable compounds (Table 1). With the increase in the chitosan viscosity and its content in the system, the CVD permeability across the skin-simulating membrane decreased. It confirmed the results of previous studies of permeability from transdermal patches through the rat abdominal skin where CVD was released from the formulation and permeated through the rat skin. It hence could permeate through the human skin as well [50]. The potential use of CVD-chitosan systems in a topical application is an interesting approach that needs to be developed.

**Figure 9:**
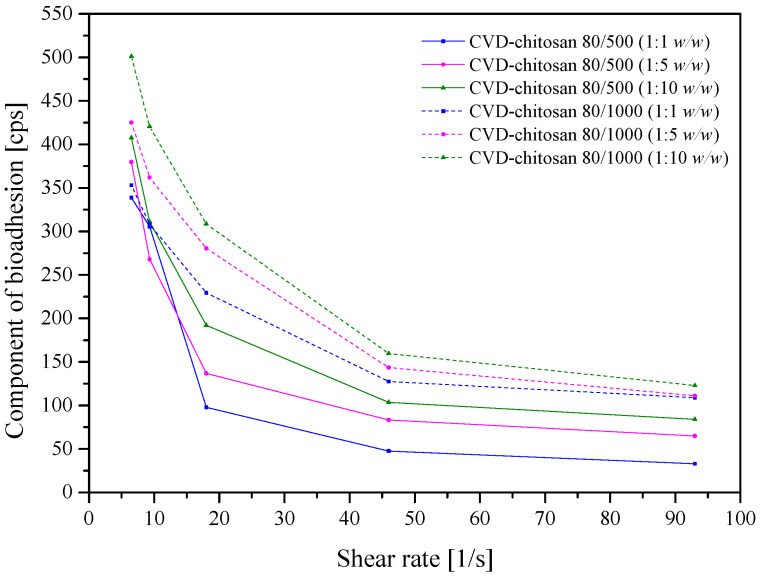
Effect of the shear rate on the component of bioadhesion of 1% CVD-chitosan systems.

Adhesive, topical delivery systems have been developed to be localized onto a biological surface. Rheological measurements evaluated the mucoadhesive properties of chitosan-based systems with CVD. Chitosan is a compound possessing amino groups that interact with negatively charged mucin chains, mainly sialic acid in mucin, demonstrating a high interaction with mucin (Figure 9) [52]. The bioadhesion increased with increasing system viscosity (chitosan 80/1000 > 80/500) and chitosan in the system. The increase in bioadhesion has both advantages, such as increased membrane adhesion and prolonged contact time of the product with the mucosa; however, it has serious disadvantages, such as the prolonged and limited release of the active compounds from the drug delivery system.

## 3. Materials and Methods

### 3.1. Materials

CVD (purity > 98%) was supplied by Sigma Aldrich (St Louis, MO, USA). HPLC grade acetonitrile was obtained by ROMIL; formic acid (purity 98–100%) and ethanol (purity > 99%) were supplied by POCH Gliwice (Gliwice, Poland). Hydrochloric acid, sodium hydroxide, and potassium dihydrogen phosphate were purchased by Avantor Performance Materials Poland S.A (Deventer, The Netherlands). Chitosan 80/500 (degree of deacetylation: 77.6–82.5%; viscosity: 351–750 mPas; molecular weight 200–400 kDa) and 80/1000 (degree of deacetylation: 77.6–82.5%; viscosity: 751–1250 mPas; molecular weight 200–500 kDa) were supplied by Heppe Medical Chitosan GmbH (Halle, Germany). Prisma™ HT buffer, Acceptor Sink Buffer, G.I.T. lipid solution were obtained by Pion Inc. (Billerica, MA, USA). High-quality pure water and ultra-high-quality pure water were prepared by using a Direct-Q 3 UV Merck Millipore purification system (Merck, Warsaw, Poland).

### 3.2. Development and Validation of HPLC Method to Determine the Content of the CVD

The CVD identity and CVD concentrations in the samples collected from dissolution studies were determined by using the UHPLC Diode Array Detection. The separation of CVD was possible using the LC system (DionexThermolineFisher Scientific, Waltham, MA, USA) equipped with Chromeleon software version 7.0. Separations were performed on a Kinetex-C18 column (100 mm × 2.1 mm, 2.6 μm). The CVD detection was performed using a diode array detector at a wavelength maximum (*λ*_max_) of 240 nm. The mobile phase consisted of a mixture of acetonitrile and 0.1% formic acid (50:50 *v*/*v*) with a mobile phase flow rate of 0.3 mL/min. The column and autosampler tray were set at 25 °C. The UHPLC-DAD method was validated according to the International Conference on Harmonization Guideline Q2 in regard to selectivity, linearity, intra- and inter-day precision, limits of detection (LOD) and quantitation (LOQ).

### 3.3. Preparation of Solid Samples of Carvedilol with Chitosan

The carvedilol (CVD) was mixed in an agate mortar for 1 h with two chitosans of different viscosity 80/500 and 80/1000 in weight ratio 1:1 (*w*/*w*), 1:5 (*w*/*w*), and 1:10 (*w*/*w*) to obtain a uniform powder. To avoid losses in creating systems, due to the hygroscopicity of CVD, a wetting substance was used (0.5 mL of 96% ethanol for every 100 mg of the system). The obtained powder was stored in a refrigerator at 6 °C tightly closed vial with limited air access.

Six CVD-chitosan systems were obtained:

(1) CVD-chitosan 80/500 (1:1 *w*/*w*)
(2) CVD-chitosan 80/500 (1:5 *w*/*w*)
(3) CVD-chitosan 80/500 (1:10 *w*/*w*)
(4) CVD-chitosan 80/1000 (1:1 *w*/*w*)
(5) CVD-chitosan 80/1000 (1:5 *w*/*w*)
(6) CVD-chitosan 80/1000 (1:10 *w*/*w*).

### 3.4. Powder X-ray Diffraction (PXRD)

PXRD was conducted at room temperature using an RPANalitycal Empyrean (Malvern Panalytical, Malvern, UK) equipped with a Cu Kα (1.54056 Å) radiation X-ray source at 45 kV and 40 mA. The samples were mounted on a low background silicon sample holder and scanned over a range of 3–50° 2*θ* with a step width of 0.017° 2*θ* and signal collection time of 15 s per step.

### 3.5. Fourier Transform Infra-Red (FTIR) Spectroscopy

FTIR spectra of the samples were obtained with an FT-IR Bruker IFS 66 v/S (Bruker, Bremen, Germany) equipped with a Bruker Hyperion1000 microscope (Bruker, Bremen, Germany) using IR grade KBr mixed with the samples at a ratio of 1:100 (*w*/*w*). The pellets were prepared by applying 8 metric tonnes of pressure in a hydraulic press. The vibrational infrared spectra were measured between 400 and 4000 cm^−1^. To analyze changes in positions and intensity of bands in experimental spectra of CVD-chitosan systems, quantum chemical calculations were performed based on B3LYP functional and 6–31G(d,p) as a basis set. All the calculations were made by using the Gaussian 09 package and the GaussView application.

### 3.6. Raman Spectroscopy

Raman scattering spectra were recorded with a Raman FRA106/S spectrometer with laser excitation λ_exc_ = 785 nm (HeANe laser). The spectra were measured between 200 and 2000 cm^−1^. In each case, the laser beam’s power at the sample was less than 1 mW to avoid the sample’s damages.

### 3.7. Differential Scanning Colorimetry (DSC) and Thermogravimetric Analysis (TGA)

DSC thermograms were recorded using a DSC 214 Polyma (Netzsch). Accurately weighed samples were placed in sealed cells and heated at a scanning rate of 10 °C/min from 25 °C to 290 °C under a nitrogen purge gas with a flow rate of 20 mL/min.

TGA analyses were performed by using a TG 209 F3 Tarsus (Netzsch). Samples were heated at a scanning rate of 10 °C/min from 35 °C to 600 °C under a nitrogen purge gas with a flow rate of 20 mL/min.

### 3.8. Scanning Electron Microscope (SEM) Images

SEM images were processed using the scanning electron microscope (SEM, VEGA 5135, Tescan, Brno, Czech Republic) with the energy dispersive spectrometer (EDS, PTG Prison Avalon, Princeton Gamma Tech., Princeton, NY, USA) at 11.0 kV. Due to the low conductivity, the samples were sprayed with a carbon layer before the analysis in order to improve the resolution of the obtained photos. Pictures at a 1000-fold magnification are shown.

### 3.9. Dissolution Studies

The change in dissolution rate of the obtained powder CVD combinations was examined by determining the dissolution rate profiles following the requirements of the European Pharmacopoeia at 37 ± 0.5 °C, using a paddle apparatus (Agilent, Santa Clara, CA, USA) with a paddle rotation speed of 50 rpm. As the acceptor dissolution media, 500 mL of 0.1 mol/L hydrochloric acid (pH ~ 1.2) and phosphate buffer (pH ~ 6.8) were used in the pH range corresponding to the gastrointestinal tract environment. CVD (25 mg) and CVD-chitosan systems (weight corresponding to the content of 25 mg of carvedilol) were weighed into gelatin capsules and placed in a sinker to prevent flotation of the capsule on the liquid’s surface. Dissolution samples were taken at appropriate time points, and an equal volume of temperature-equilibrated media was replaced. The samples were filtered through 0.45 μm nylon membrane filters.

Dissolution profiles were compared using the model proposed by Moore and Flanner, which is based on two-factor values, difference *f*_1_ and similarity *f*_2_ [53], according to the formulas below:

$$f_{1}=\frac{\sum_{j=1}^{n}\left|R_{j}-T_{j}\right|}{\sum_{j=1}^{n}R_{j}}\times 100$$

$$\;f_{2}=50\times \log\left({\left(1+\left(\frac{1}{n}\right)\overset{n}{\underset{j=1}{\sum }}{\left|R_{j}-T_{j}\right|}^{2}\right)}^{-\frac{1}{2}}\times 100\right)$$

where *n* is the sampling number, *R_j_* and *T_j_* are the percent dissolved of the reference (CVD) and test products (CVD-chitosan systems) at each time point.

Release profiles are assumed to be similar when *f*_1_ is between 0 and 15, while *f*_2_ is close to 100 (not less than 50).

### 3.10. Permeability Studies

The permeability of CVD and their chitosan systems was investigated through the artificial biological membrane using a PAMPA (Pion Inc., Billerica, MA, USA), a model simulating the gastrointestinal tract (GIT-PAMPA). The model consists of 96-well plates assembled in a “sandwich” separated from each other by a 120-μm-thick microfilter disc coated with a 20% (*w*/*v*) dodecane solution of a lecithin mixture. Donor solutions with a CVD concentration of 25 mg/1000 mL corresponded to gastric and intestinal pH. Plates were incubated for 3 h at 37 °C with continuous shaking at 50 rpm corresponding to paddle speed during a dissolution study.

Apparent permeability coefficients (*P_app_*) were used to assess the permeability of the CVD and CVD-chitosan systems and were calculated from the following equation:

$$P_{app}=\frac{-ln\left(1-\frac{C_{A}}{C_{equilibrium}}\right)}{S\times \left(\frac{1}{V_{D}}+\frac{1}{V_{A}}\right)\times t}$$

where *V_D_*—donor volume, *V_A_*—acceptor volume, *C_equilibrium_*—equilibrium concentration $C_{equilibrium}=\frac{C_{D}\times V_{D}+C_{A}\times V_{A}}{V_{D}+V_{A}}$, *C_D_*—donor concentration, *C_A_*—acceptor concentration, *S*—membrane area, *t*—incubation time (in seconds).

To verify that *P_app_* determined for permeability was statistically different, an ANOVA test was used. Compounds with *P_app_* < 1 × 10^−6^ cm/s are classified as low-permeable and those with *P_app_* > 1 × 10^−6^ cm/s as high-permeable compounds [39].

If the transdermal application was considered, it was necessary to check the skin permeability of the drug. The skin permeability of CVD and chitosan-based systems was obtained by using the Skin-PAMPA system. This system is built up as the system described above. Solutions of the drug (25 mg/1000 mL) and its systems were fully dissolved in a buffer at pH 6.5 and added to the donor compartments, corresponding to the skin environment. The acceptor solution was adjusted to pH 7.4. The plates were put together and incubated at 32 °C for 240 min in a humidity-saturated atmosphere. The CVD concentrations were determined by using the UV spectrophotometer (*λ*_max_ = 240 nm).

The apparent permeability coefficient (*P_app_*) was calculated by using the equation given above.

### 3.11. In Vitro Assessment of Mucin–Chitosan Bioadhesive Bond Strength

Due to the fact that mucins, high molecular weight glycoproteins, occurs in normal skin in small amounts, a viscometric method was used to quantify mucin–chitosan bioadhesive bond strength. The assessment was performed according to the method described by Hassan and Gallo [54]. Dried mucin was hydrated with 0.1 mol/L acetate buffer by gentle stirring for 3 h at room temperature to obtain a dispersion of 20% (*w*/*v*). Powders CVD-chitosan systems in concentration 1% were also dissolved in 0.1 mol/L acetate buffer. 6 mL of mucin dispersion (20% *w*/*v*) were mixed with 2 mL of powder systems for 15 min. Viscosities of CVD-chitosan systems were measured at room temperature at the shear rate in the range 9.3–93.0 s^−1^ by using Brookfield DV2T Viscometer.

The viscosity coefficient of a hydrophilic dispersion containing mucin and a bioadhesive polymer was calculated from the following equation:

η_t_ = η_m_ + η_p_ + η_b_

where η_t_ is the viscosity coefficient of the system, and η_m_ and η_p_ are the individual viscosity coefficients of music and bioadhesive polymer, respectively, and η_b_ is the viscosity of component due to bioadhesion and can be obtained by rearranging above equation:

η_b_ = η_t_ − η_m_ − η_p_

The force of bioadhesion F represented the additional intermolecular frictional force per unit area and was determined by:

F = η_b_σ

where σ is the rate of shear per second.

## 4. Conclusions

Preparation of the CVD-chitosan system by kneading allows obtaining a physical mixture in which CVD’s physicochemical properties essential for the functioning of sustained-release delivery systems were modified. The addition of chitosan in weight ratio 1:1 and 1:5 resulted in improved CVD solubility in the environment simulating the stomach–intestine as well as skin conditions. Chitosan did not adversely affect the cell membranes’ model system’s permeability, both in the gastrointestinal tract and in the skin model. The bioadhesion study showed better properties of chitosan 80/1000; in both cases, increasing the amount of chitosan in the system increased the viscosity, improving the system’s bioadhesion.

The conducted research and the obtained results indicate a significant potential of using chitosan as an excipient in modern drug delivery systems of CVD.

## Figures and Tables

**Table 1:** Calculated apparent permeability coefficients of CVD and CVD-chitosan systems for GIT-PAMPA and Skin-PAMPA tests.

CVD Systems	GIT-PAMPA		Skin-PAMPA
	pH 1.2	pH 6.8	
	Apparent Permeability Coefficients *P_app_* × 10^−6^ [cm/s]		
CVD	13.8 ± 0.01	20.9 ± 0.02	83.30 ± 8.62
CVD-chitosan 80/500 (1:1 *w*/*w*)	9.75 ± 0.04	15.6 ± 0.03	98.50 ± 11.33
CVD-chitosan 80/500 (1:5 *w*/*w*)	17.6 ± 0.04	21.3 ± 0.03	75.50 ± 15.78
CVD-chitosan 80/500 (1:10 *w*/*w*)	24.0 ± 0.02	16.5 ± 0.02	58.87 ± 14,87
CVD-chitosan 80/1000 (1:1 *w*/*w*)	19.7 ± 0.03	12.6 ± 0.04	90.54 ± 8.42
CVD-chitosan 80/1000 (1:5 *w*/*w*)	7.39 ± 0.01	21.6 ± 0.03	72.90 ± 9.55
CVD-chitosan 80/1000 (1:10 *w*/*w*)	10.06 ± 0.01	8.47 ± 0.02	53.30 ± 15.13

## Data Availability

Data is contained within the article and Supplementary Materials.

## Supplementary Materials

The following are available online at https://www.mdpi.com/article/10.3390/ph14080712/s1, Figure S1: TGA curves of the thermal degradation of CVD and the solid systems of: CVD-chitosan 80/500 (a) and CVD-chitosan 80/1000 (b), Table S1: Validation parameters of UHPLC-DAD method, Table S2: Values of coefficients *f*1 and *f*2 at pH 1.2, Table S3: Values of coefficients *f*1 and *f*2 at pH 6.8.
