# Supplementary material for: Chitosan as Valuable Excipient for Oral and Topical Carvedilol Delivery Systems
Source: Pharmaceuticals (Basel). 2021 Jul 23;14(8):712. doi: 10.3390/ph14080712 (PMC8401298; doi:10.3390/ph14080712)

SUPPLEMENTARY MATERIALS

Table S1. Validation parameters

Parameter	Solvent: acetic acid
Linearity: y = ax + b	y=3273,94x – 3,3772
a ± S_a	15,892
b ± S_b	0,2563
Correlation coefficient (*r*)	0,9999
Range of linearity [µg/ml]	0,0026-0,0500
Limit of detection (LOD): LOD = 3,3 SD/a [µg/ml]	1,2099 x 10^{-4}
Limit of quantification (LOQ): LOQ = 10 SD/a [µg/ml]	3,6670 x 10^{-4}
Intra-day precision, RSD	
0,0026 µg/ml	1,32 %
0,0156 µg/ml	1,02 %
0,026 µg/ml	1,96 %
Inter-day precision, RSD	
0,0026 µg/ml	1,35 %
0,0156 µg/ml	1,32 %
0,026 µg/ml	1,89 %
Accuracy	99,52 %

Table S2. Values of coefficients f_1 oraz f_2 at pH 1.2

	Carvedilol	CVD-chitosan 80/500 (1:1 *w/w*)	CVD-chitosan 80/500 (1:5 *w/w*)	CVD-chitosan 80/500 (1:10 *w/w*)	CVD-chitosan 80/1000 (1:1 *w/w*)	CVD-chitosan 80/500 (1:5 *w/w*)	CVD-chitosan 80/1000 (1:1 *w/w*)
Carvedilol	x	f_1=40,19<br>f_2=32,95	f_1=19,53<br>f_2=51,23	f_1=13,07<br>f_2=59,79	f_1=28,73<br>f_2=42,92	f_1=16,91<br>f_2=54,32	f_1=25,35<br>f_2=45,63
CVD-chitosan 80/500 (1:1 *w/w*)	f_1=40,19<br>f_2=32,95	X	f_1=14,4<br>f_2=51,76	f_1=32,46<br>f_2=34,24	f_1=4,43<br>f_2=76,19	f_1=12,54<br>f_2=54,74	f_1=48,44<br>f_2=28,43
CVD-chitosan 80/500 (1:5 *w/w*)	f_1=19,53<br>f_2=51,23	f_1=14,4<br>f_2=51,76	x	f_1=25,27<br>f_2=41,83	f_1=13,16<br>f_2=55,87	f_1=14,71<br>f_2=53,49	f_1=36,31<br>f_2=33,99
CVD-chitosan 80/500 (1:10 *w/w*)	f_1=13,07<br>f_2=59,79	f_1=32,46<br>f_2=34,24	f_1=25,27<br>f_2=41,83	x	f_1=44,11<br>f_2=36,09	f_1=30,87<br>f_2=43,80	f_1=15,34<br>f_2=54,80
CVD-chitosan 80/1000 (1:1 *w/w*)	f_1=28,73<br>f_2=42,92	f_1=4,43<br>f_2=76,19	f_1=13,16<br>f_2=55,87	f_1=44,11<br>f_2=36,09	x	f_1=9,71<br>f_2=56,77	f_1=40,85<br>f_2=25,77
CVD-chitosan 80/1000 (1:5 *w/w*)	f_1=16,91<br>f_2=54,32	f_1=12,54<br>f_2=54,74	f_1=14,71<br>f_2=53,49	f_1=30,87<br>f_2=43,80	f_1=9,71<br>f_2=56,77	x	f_1=34,88<br>f_2=31,28
CVD-chitosan 80/1000 (1:10 *w/w*)	f_1=25,35<br>f_2=45,63	f_1=48,44<br>f_2=28,43	f_1=36,31<br>f_2=33,99	f_1=15,34<br>f_2=54,80	f_1=40,85<br>f_2=25,77	f_1=34,88<br>f_2=31,28	x

Table S3. Values of coefficients f_1 oraz f_2 at pH 6.8

	Carvedilol	CVD-chitosan 80/500 (1:1 *w/w*)	CVD-chitosan 80/500 (1:5 *w/w*)	CVD-chitosan 80/500 (1:10 *w/w*)	CVD-chitosan 80/1000 (1:1 *w/w*)	CVD-chitosan 80/500 (1:5 *w/w*)	CVD-chitosan 80/1000 (1:1 *w/w*)
Carvedilol	x	f_1=87,16<br>f_2=30,56	f_1=120,11<br>f_2=23,55	f_1=31,04<br>f_2=51,3	f_1=49,08<br>f_2=43,88	f_1=108,81<br>f_2=25,96	f_1=19,10<br>f_2=61,81

CVD-chitosan 80/500 (1:1 *w/w*)	f_1=87,16<br>f_2=30,56	X	f_1=17,78<br>f_2=50,91	f_1=32,45<br>f_2=39,28	f_1=69,05<br>f_2=22,56	f_1=12,66<br>f_2=59,04	f_1=40,13<br>f_2=34,37
CVD-chitosan 80/500 (1:5 *w/w*)	f_1=120,118<br>f_2=23,55	f_1=17,78<br>f_2=50,91	x	f_1=14,97<br>f_2=51,38	f_1=73,72<br>f_2=17,38	f_1=5,66<br>f_2=17,38	f_1=49,17<br>f_2=26,09
CVD-chitosan 80/500 (1:10 *w/w*)	f_1=31,04<br>f_2=51,3	f_1=32,45<br>f_2=39,28	f_1=14,97<br>f_2=51,38	x	f_1=54,63<br>f_2=35,61	f_1=65,12<br>f_2=32,39	f_1=13,22<br>f_2=64,93
CVD-chitosan 80/1000 (1:1 *w/w*)	f_1=49,08<br>f_2=43,88	f_1=69,05<br>f_2=22,56	f_1=73,72<br>f_2=17,38	f_1=54,63<br>f_2=35,61	x	f_1=261,09<br>f_2=19,17	f_1=95,22<br>f_2=40,93
CVD-chitosan 80/1000 (1:5 *w/w*)	f_1=108,81<br>f_2=25,9	f_1=12,66<br>f_2=59,04	f_1=5,66<br>f_2=70,8	f_1=65,12<br>f_2=32,39	f_1=261,09<br>f_2=19,17	x	f_1=86,63<br>f_2=28,82
CVD-chitosan 80/1000 (1:10 *w/w*)	f_1=19,10<br>f_2=61,81	f_1=40,13<br>f_2=34,37	f_1=49,17<br>f_2=26,09	f_1=13,22<br>f_2=64,93	f_1=95,22<br>f_2=40,93	f_1=86,63<br>f_2=28,82	x

Figure S1. Chromatogram of carvedilol in the developed HPLC method

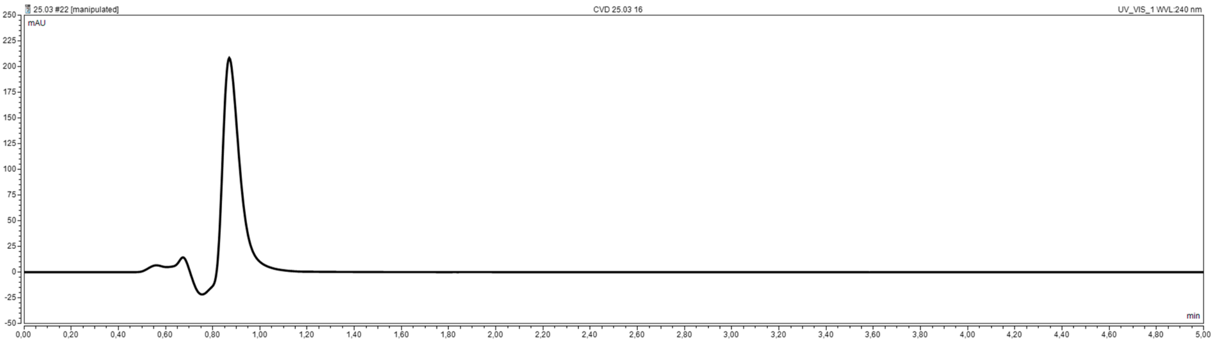

Figure S2. TG curves of CVD and CVD-chitosan 80/500 systems

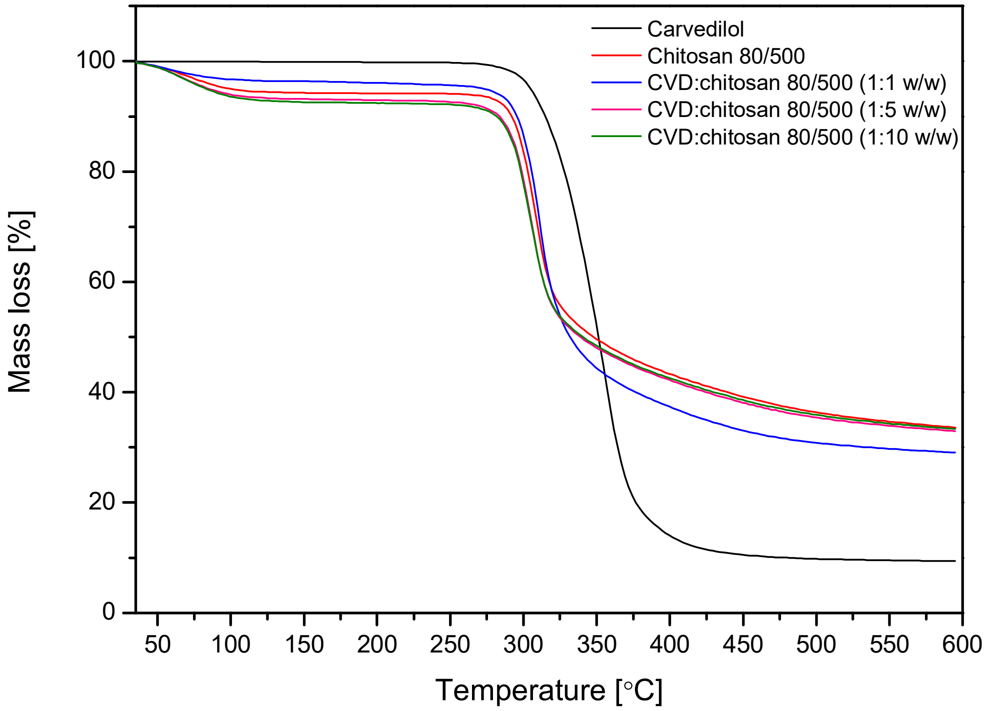

Figure S3. TG curves of CVD and CVD-chitosan 80/1000 systems

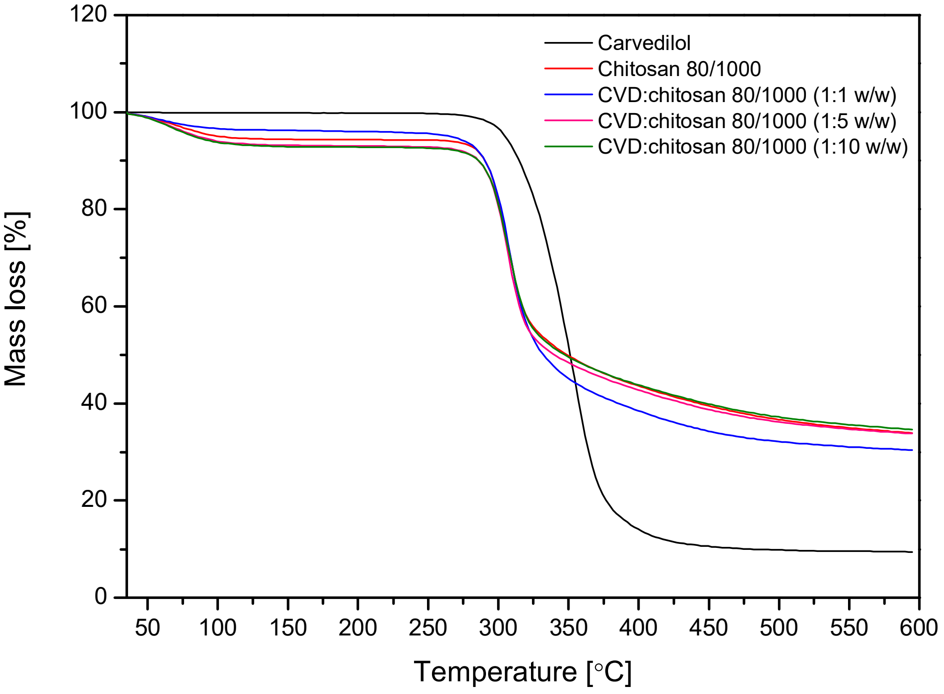

Supplement: Supplementary file 1 [file pharmaceuticals-14-00712-s001.zip › pharmaceuticals-1281457-supplementary.pdf]
